# HMGB1 in nervous system diseases: A common biomarker and potential therapeutic target

**DOI:** 10.3389/fneur.2022.1029891

**Published:** 2022-10-31

**Authors:** Di Mao, Yuan Zheng, Fenfen Xu, Xiao Han, Hongyang Zhao

**Affiliations:** ^1^Department of Pediatrics, Jinan Central Hospital, Shandong University, Jinan, China; ^2^Department of Pediatrics, Central Hospital Affiliated to Shandong First Medical University, Jinan, China

**Keywords:** HMGB1, biomarker, nervous system diseases, neuroinflammation, therapeutic target

## Abstract

High-mobility group box-1 (HMGB1) is a nuclear protein associated with early inflammatory changes upon extracellular secretion expressed in various cells, including neurons and microglia. With the progress of research, neuroinflammation is believed to be involved in the pathogenesis of neurological diseases such as Parkinson's, epilepsy, and autism. As a key promoter of neuroinflammation, HMGB1 is thought to be involved in the pathogenesis of Parkinson's disease, stroke, traumatic brain injury, epilepsy, autism, depression, multiple sclerosis, and amyotrophic lateral sclerosis. However, in the clinic, HMGB1 has not been described as a biomarker for the above-mentioned diseases. However, the current preclinical research results show that HMGB1 antagonists have positive significance in the treatment of Parkinson's disease, stroke, traumatic brain injury, epilepsy, and other diseases. This review discusses the possible mechanisms by which HMGB1 mediates Parkinson's disease, stroke, traumatic brain injury, epilepsy, autism, depression, multiple sclerosis, amyotrophic lateral sclerosis, and the potential of HMGB1 as a biomarker for these diseases. Future research needs to further explore the underlying molecular mechanisms and clinical translation.

## Introduction

**Figure 1:**
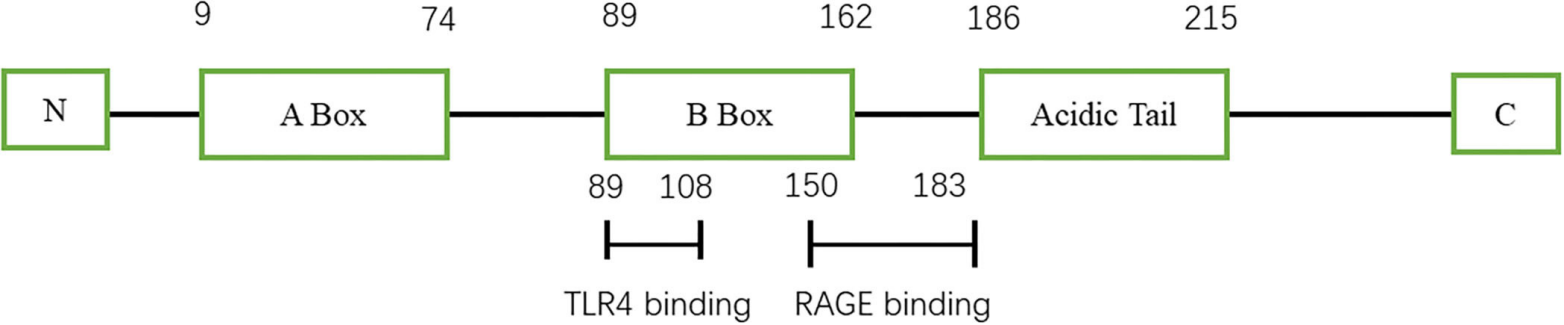
Schematic view of HMGB1 structure.

**Figure 2:**
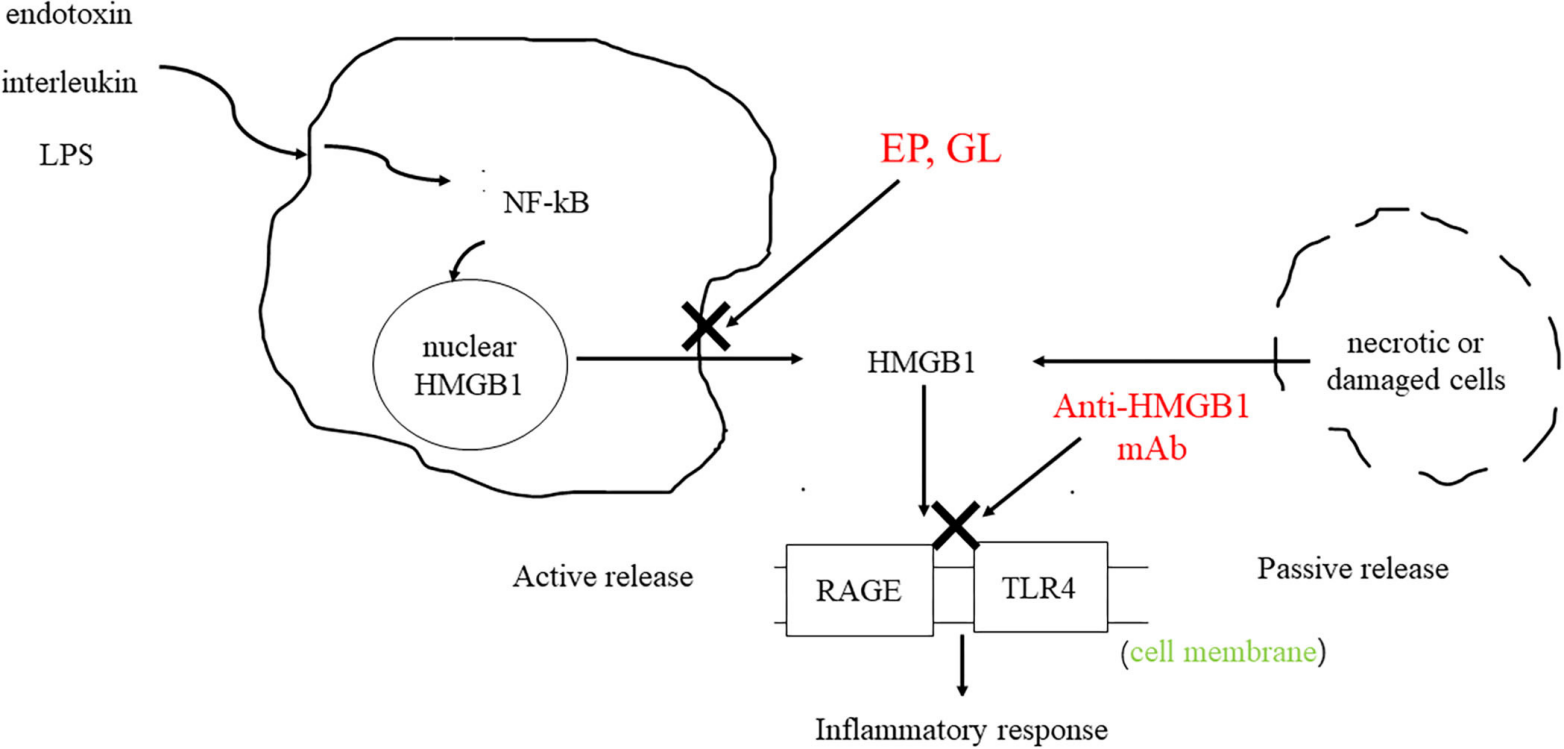
HMGB1 can be actively secreted by immune cells or passively secreted by necrotic or damaged cells. HMGB1 binds to RAGE and TLR4 to activate downstream signaling pathways, resulting in the upregulation of cytokines by pro-inflammatory cells. As HMGB1 antagonists, GL and EP can inhibit the release of HMGB1. The anti-HMGB1 mAb neutralizes HMGB1.

High-mobility group protein (HMG) was first discovered in bovine thymus in 1973 (1). Subsequent studies found that inhibition of high-mobility group-1 (HMG-1) protein could reduce the mortality of patients with sepsis, thus confirming the role of HMG-1 as an inflammatory factor (2). In 2000, Bustin (3) systematically classified the HMG family and divided them into three categories: high-mobility group-A (HMGA), high-mobility group box (HMGB), and high-mobility group-N (HMGN) according to their functions. Among them, HMGB was further divided into HMGB1, HMGB2, and HMGB3. HMGB1 is composed of three domains, including two DNA-binding domains (A box and B box) and an acidic tail (Figure 1) (4). Both A box and B box are composed of three α-helix structures, which can interact with deoxyribonucleic acid (DNA) nonspecifically (5). HMGB1 has two nuclear localization sequences (NLSs) located between the A box (aa 28–44) and the B box and C-terminal tail (aa 179–185) (6). When immune cells respond to endogenous or exogenous stimuli such as endotoxin, interleukin, and hypoxia, HMGB1 can be actively released (7). Meanwhile, necrotic or damaged cells can passively release HMGB1 (8). In addition, phagocytosis of apoptotic cells by macrophages can lead to the further release of HMGB1 (9). HMGB1 utilizes various membrane receptors during its signaling cascade. Among the numerous HMGB1 extracellular receptors, the receptor for advanced glycation end products (RAGE) and toll-like receptor 4 (TLR4) are the widely studied and reported receptors. Binding to RAGE occurs at residues 150–183 of the molecule, while TLR4 binding occurs at residues 89–108 of the HMGB1 B box (6). HMGB1 binds to receptors such as TLR4 and RAGE and leads to the upregulation of cytokines by pro-inflammatory cells by activating nuclear factor kappa-light-chain-enhancer of activated B cells (NF-κB) and mitogen-activated protein (MAP) kinase signaling pathways (Figure 2).

Interestingly, overexpression of extracellular HMGB1 has been observed in clinical and preclinical studies on Parkinson's disease (10), stroke (11), traumatic brain injury (12), epilepsy (13), autism (14), depression (15), multiple sclerosis (16), and amyotrophic lateral sclerosis (17). Although relevant clinical studies are lacking, positive structures have been achieved in animal models based on HMGB1 antagonists (anti-HMGB1 monoclonal antibody, ethyl pyruvate, and glycyrrhizin) targeting extracellular HMGB1 therapy. However, there are different isoforms of HMGB1, fully reduced (frHMGB1) and disulfide HMGB1 (dsHMGB1), which are thought to bind to the receptor and can play a pro-inflammatory role, and fully oxidized HMGB1(oxHMGB1) is inert (5). The fact that a mixture of different HMGB1 isoforms is present in the extracellular matrix challenges determining the exact role of individual antagonists. The preclinical and clinical evidence discussed here reinforces HMGB1 as a promising candidate as a common biomarker and therapeutic target for neurological disorders in which neuroinflammatory pathways play a central role.

## Parkinson's disease

Parkinson's disease is the second most common neurodegenerative disorder in the elderly, mainly manifested by resting tremor, bradykinesia, rigidity, and postural reflex abnormalities. Parkinson's disease (PD) is pathologically characterized with loss of dopamine (DA) neurons in the midbrain substantia nigra pars compacta (SNpc) (18) and α-synuclein (α-syn) containing Lewy bodies (LBs) formation (19). The pathogenesis of PD is currently unclear. Recent studies have found that elevated levels of HMGB1 protein were detected in postmortem midbrain tissue as well as cerebrospinal fluid (CSF) and serum of PD patients. At the same time, it was found that HMGB1 protein is mainly located in the cytoplasm of PD patients and in the nucleus of control patients, which may indicate that HMGB1 translocation is involved in the pathogenesis of PD (20). In Parkinson's disease, HMGB1 specifically binds to α-syn aggregated in LBs isolated from rat brain, suggesting a promoting role of HMGB1 in neurodegenerative processes in the chronic phase of the disease (21). Extracellular α-syn aggregates can activate astrocytes or microglia, leading to persistent inflammation and subsequent neurodegeneration (22). In primary cultures of mouse neurons and glial cells, HMGB1 was found to bind to the microglial membrane receptor macrophage antigen complex 1 (Mac1) and activate the NF-κB pathway and nicotinamide adenine dinucleotide phosphate (NADPH) oxidase expression, thereby inducing pro-inflammatory factor expression and neurotoxicity. Furthermore, the HMGB1-Mac1 interaction reduces dopamine uptake and the number of dopaminergic neurons (23). On the contrary, the translocation of HMGB1 from the nucleus to the cytoplasm leads to the binding of HMGB1 to Beclin1 to dissociate Beclin1-B-cell lymphoma (Bcl-2) and induce autophagy (24), promoting the self-clearance of α-syn (24, 25), thereby delaying disease progression. Experiments on the PC12 cell line confirmed that inhibition of HMGB1 translocation inhibits autophagy, resulting in the accumulation of α-syn that exacerbates neuronal damage (26). In a 1-methyl-4-phenyl-1,2,3,6-tetrahydropyridine (MPTP)-induced mouse model of acute Parkinson's disease, HMGB1 can promote the expression of tyrosine hydroxylase (TH) in the striatum, thereby maintaining dopaminergic neuron function (27).

In view of the above, HMGB1-targeted or HMGB1/TLR4 pathway inhibition can serve as a rational approach for PD therapy and may serve as a potential biological target (Tables 1, 2). Intravenous administration of HMGB1 antibody attenuated MPTP-induced dopaminergic cell death (20) and reduced PD behavioral symptoms (28). Injection of ethyl pyruvate (EP) into a mouse subacute Parkinson's model can effectively reduce the activation of microglia and inhibit the neuroinflammation mediated by microglia (29). These results are consistent with another study showing that intravenous injection of anti-HMGB1 monoclonal antibodies (mAbs) in a rat PD model significantly inhibited microglial activation and reduced the loss of dopaminergic neurons in SNpc (33). Furthermore, the anti-HMGB1 treatment group inhibited the disruption of the blood–brain barrier (BBB) and the increase in vascular permeability caused by 6-hydroxydopmaine (6-OHDA) neurotoxicity (28). At present, the conventional methods for the clinical treatment of PD are limited, and the targeted therapy of HMGB1 provides a possible idea. However, long-term efficacy and safety in humans have not been studied. At the same time, the related side effects of HMGB1-targeted therapy should also be alerted. However, glycyrrhizin (GL) may lead to complications, such as hypertension and hypokalemia (34). EP is a non-specific HMGB1 inhibitor that inhibits the release of HMGB1 only in live cells, but not in dead cells (35). Long-term use of antibodies may also lead to autoimmune and hematological diseases (28).

**Table 1:** Studies targeting HMGB1 in PD.

**S.N**.	**Study model**	**Treatment**	**Mode of inhibition**	**Observations**	**References**
1	MPTP-induced PD mouse model	Anti-HMGB1 mAb	Neutralization	Inhibits dopaminergic cell death and reduces RAGE and TNF-α levels	(20)
2	6-OHDA-induced PD mouse model	Anti-HMGB1 mAb	Neutralization	inhibits the activation of microglia, the destruction of the BBB and the expression of IL-1β and IL-6 Reduced PD behavioral symptoms	(28)
3	MPTP-induced PD mouse model	EP	Release inhibition	Restoration of dopaminergic neuron numbers in substantia nigra and striatum	(29)
4	MPTP-induced PD zebrafish larvae model	GL	Release inhibition	Increases the length of DA neurons in the zebrafish brain and reduces the number of apoptotic cells in the zebrafish brain	(30)

**Table 2:** Studies targeting HMGB1/TLR4 pathway in PD.

**S.N**.	**Study model**	**Treatment**	**Mechanism**	**Observations**	**References**
1	MPTP-induced PD mouse model	OMT	Inhibition of HMGB1/TLR4/NF-κB pathway	Inhibits HMGB1/TLR4/NF-κB signaling pathway Attenuates microglia-mediated neuroinflammatory responses Dose-dependently attenuates MPTP-induced dyskinesia	(31)
2	ROT-induced PD mouse model	ALO	Inhibition of HMGB1/TLR4/NLRP3 pathway	Inhibits striatal microglial activation	(32)

## Stroke

Stroke is one of the leading causes of disability and death, and its pathophysiology is complex. Neuroinflammation, oxidative stress, and apoptosis are thought to be involved in the occurrence and development of stroke (36). Neuronal HMGB1 release is increased in stroke models. Zhang et al. (37) found elevated levels of HMGB1 in the CSF of an animal model of cerebral ischemia. During ischemic stroke (IS), HMGB1 may signal through its possible receptors, such as RAGE, toll-like receptors (TLRs), and matrix metalloproteinases (MMPs) (38). Studies have found that HMGB1 translocation is very sensitive to hypoxia, and it is released from the nucleus early in stroke to function as a pro-inflammatory factor (39, 40). Animal studies have found that HMGB1 is translocated from the nucleus to the cytoplasm of the peri-infarct cortical region 2 h after ischemia–reperfusion (41). Another study yielded the same results that HMGB1 was released from the nucleus into the cytoplasm of the ipsilateral brain 1 h after intracerebral hemorrhage (ICH) induction, possibly as an early pro-inflammatory mediator promoting neuroinflammation within the neurovascular unit (42). In addition, HMGB1 can increase the level of glutamate leading to excitotoxicity (43). PC12 cells exposed to oxygen-glucose deprivation (OGD) increased HMGB1 secretion and induced cell death in a dose-dependent manner (44, 45). Furthermore, there was a correlation between extracellular HMGB1 levels and stroke severity in the rat middle cerebral artery occlusion (MCAO) model. Higher levels of extracellular HMGB1 in serum and cerebrospinal fluid were associated with larger infarct volume (46) and more severe disease (11).

Anti-HMGB1 antibody can significantly reduce the size of cerebral infarction in rats and improve the symptoms of neurological deficit (47). In addition, studies have found that anti-HMGB1 antibodies can protect the BBB, reduce circulating HMGB1, and at the same time reduce brain edema (48). Anti-HMGB1 mAbs treatment inhibited neuronal translocation and release of HMGB1 itself, suggesting the existence of a positive feedback loop between HMGB1 mobilization and brain inflammatory responses (49). Short hairpin RNA-mediated HMGB1 (ShHMGB1) can reduce the infarct size in the rat MCAO model, which may be caused by shHMGB1 reducing HMGB1 expression in the acute phase (39). GL, a natural inhibitor of HMGB1, potently inhibits MMP-9 activity, protects tight junction claudin-5 and extracellular matrix collagen IV, and preserves BBB integrity in the brain of delayed tissue plasminogen activator (t-PA)-treated ischemia–reperfusion rats. In addition, in the setting of delayed t-PA treatment, GL reduces mortality, neurological deficit scores, and brain edema in MCAO brains (48). In contrast, in a rodent ICH model, HMGB1-RAGE signaling appears to upregulate vascular endothelial growth factor (VEGF) expression and promote angiogenesis in the late post-ICH period (50). In conclusion, HMGB1 may be involved in the pathophysiology of stroke, but animal experiments have shown that HMGB1 has a biphasic effect in stroke patients, and it is unclear to what extent it promotes the development of the disease. However, elevated levels of HMGB1 within the first 24 h after ischemic stroke are considered to be a good predictor of stroke severity and clinical outcome (51), thus serving as a potential therapeutic target (Table 3). However, how to inhibit the harmful form of HMGB1 while retaining its vascular remodeling function presents new challenges for future research.

**Table 3:** Studies targeting HMGB1 in stroke.

**S.N**.	**Study model**	**Treatment**	**Mode of inhibition**	**Observations**	**References**
1	MACO-induced stroke mouse model	GL	Release inhibition	Reduces the mortality of t-PA delayed treatment of ischemic stroke model rats, reduce hemorrhagic transformation, brain swelling, BBB damage, neuronal apoptosis, and improve neurological function Inhibits ONOO-/HMGB1/TLR2 signaling pathway	(48)
2	MACO-induced stroke mouse model	Berberine	Release inhibition	Dose-dependently inhibits nuclear-cytoplasmic translocation of HMGB1 protein Inhibits HMGB1/TLR4/NF-κB signaling pathway	(52)
3	MACO-induced stroke mouse model	SSA	Release inhibition	Inhibits the release of HMGB1 in the nucleus	(53)
4	MACO-induced stroke mouse model	HP	Neutralization	Binds HMGB1 Inhibits activation of macrophages/microglia	(54)
5	MACO-induced stroke mouse model	Anti-HMGB1 antibody	Neutralization	–	(55)

## Traumatic brain injury

Traumatic brain injury (TBI) is a global public health problem, and severe TBI is characterized with high mortality (56). Neuroinflammation plays an important role in the pathological process of TBI. One study found that plasma HMGB1 levels in TBI patients were significantly higher than those in healthy controls, and HMGB1 could be used as a predictor of TBI 1-year survival (57). Animal studies found that 30 min after TBI, HMGB1 staining disappeared from the core of the contused area and was transferred to the cytoplasm at the edge of the contused area (58). Another study validated this finding by detecting HMGB1 in the cytoplasm of glial cells 4 h after TBI (59). The translocation indicated the functional activity of HMGB1 as an inflammatory mediator. However, the release of HMGB1 was age-dependent, with increases in extracellular HMGB1 in both the lesion and the perilesional neocortex in both young (3 weeks) and adult mice (8–10 weeks). However, the elevation of HMGB1 was only statistically significant in the perilesional neocortex of adult mice (60). However, enzyme-linked immunosorbent assay (ELISA) cannot distinguish between actively and passively released HMGB1, so the detected levels of HMGB1 may be actively released by immune cells, or passively released by necrotic cells, or a combination of the two (61). TBI induces an inflammatory response in brain tissue characterized by nucleocytoplasmic translocation of HMGB1, upregulation of HMGB1/HMGB1 receptors (TLR4 and RAGE), enhanced NF-κB activation, and promotion of inflammatory factors interleukin (IL)-1β, tumor necrosis factor-α (TNF-α), and IL-6 and other inflammatory cytokines (62). The HMGB1 protein contributes to brain edema by causing a decrease in occludin, claudin-5, and zonula occludens-1 (ZO-1). HMGB1 protein was also found to increase apoptosis by increasing caspase-3 levels and decreasing bcl-2 levels and to increase oxidative damage by increasing total oxidative status (63). High HMGB1 levels may impair synaptic plasticity late in TBI (64).

Currently, the treatment of TBI patients is limited and the prognosis is poor, so it is imperative to deeply study the pathophysiology of TBI and find new therapeutic targets. The prognostic value of HMGB1 is similar to the Glasgow coma score (GCS); elevated levels of HMGB1 in the ventricular CSF are associated with poorer prognosis after TBI in children (65). This suggests that HMGB1 has potential as a TBI biomarker. Primary examples of therapeutics targeting HMGB1 include GL, EP, and anti-HMGB1 mAbs (Table 4). Animal studies have found that GL can reduce inflammation by inhibiting HMGB1 translocation, inhibiting NF-κB DNA binding activity, and reducing the expression of inflammatory cytokines (62). In addition, GL can reduce brain edema, reduce apoptosis, and improve motor function recovery after TBI. GL attenuated TBI by inhibiting HMGB1, thereby inhibiting microglia/macrophages (M1) phenotype activation and promoting microglia/macrophages (M2) phenotype activation in microglia/macrophages (66). HMGB1 A-box significantly reduces brain edema, improves cellular degeneration, reduces the expression of pro-inflammatory cytokines in post-traumatic brain injury, and improves behavioral performance in TBI mice by protecting the integrity of the BBB (67) (Table 4). The expression of HMGB1 decreased after the application of EP in TBI rats, while improving cerebral edema and reducing oxidative damage (63). As an immunonutrient, Omega-3 polyunsaturated fatty acid (omega-3 PUFA) can inhibit HMGB1 nuclear translocation and HMGB1-mediated activation of TLR4/NF-κB signaling pathway, inhibit the induced microglial activation and subsequent inflammatory response, and thus exert neuroprotective effects (70) (Table 4). However, studies have found that the serum HMGB1 concentration in adults remained relatively stable in TBI, and the serum HMGB1 concentration in children increased (60). Therefore, children may benefit more in targeting HMGB1 inhibition for the treatment of TBI-induced neuroinflammation. In conclusion, animal experiments show that HMGB1-targeted therapy is an effective treatment for TBI, which can protect the integrity of the BBB, reduce brain edema, and inhibit neuroinflammation to exert neuroprotective effects. However, current animal experiments have not proved that HMGB1-targeted therapy can improve cognitive ability, and its long-term effect still needs further research. In addition, current research suggests that disulfide bond-HMGB1 plays a major role in the process of inflammation (71). How to target and inhibit the harmful subtype of HMGB1 presents a new challenge for future clinical translation.

**Table 4:** Studies targeting HMGB1 in TBI.

**S.N**.	**Study model**	**Treatment**	**Mode of inhibition**	**Observations**	**References**
1	CCI-induced TBI mouse model	GL	Release inhibition	Improves short-term spatial memory and motor learning impairments	(12)
2	CCI-induced TBI mouse model	EP	Release inhibition	Inhibits the expression of HMGB1 and TLR4, IL-1β, TNF-α and IL-6 Improves walking ability, cerebral edema	(63)
3	CCI-induced TBI mouse model	GL	Release inhibition	Improves neurological recovery after traumatic brain injury Reduces injury volume Inhibits the release of HMGB1	(66)
4	CCI-induced TBI mouse model	HMGB1 A-box	Neutralization	The HMGB1 A-box fragment is an antagonist that competes with full-length HMGB1 receptor binding Protects the integrity of the BBB, reduces cerebral edema, reduces the expression of pro-inflammatory cytokines after brain trauma, and reverses brain damage in mice with brain trauma	(67)
5	CCI-induced TBI mouse model	ω-3 PUFA	Release inhibition	Inhibits HMGB1 nucleocytoplasmic translocation/extracellular secretion is suppressed	(68)
6	Fluid percussion -induced TBI mouse model	Anti-HMGB1 mAb	Neutralization	Inhibits the activation of microglia and the death of hippocampal neurons in the ipsilateral hemisphere rat after traumatic brain injury	(69)

## Epilepsy

Epilepsy is considered to be one of the most common neurological disorders worldwide (72). Epilepsy and the mechanism of seizures are not well understood, but inflammation is thought to be an important contributor to seizures (72). In studies on animal models of epilepsy, HMGB1 has attracted attention. Animals with active epilepsy have elevated blood levels of HMGB1 compared to healthy or well-controlled individuals (73). At the same time, a clinical study found that HMGB1 levels were proportional to the severity of epilepsy, and high levels of HMGB1 may represent an increased possibility of antiepileptic drug resistance (74). Serum HMGB1 concentration can predict seizure frequency (75). In conclusion, HMGB1 can be used as a potential biomarker to predict epilepsy recurrence and prognosis. Animal studies have found that translocation and release of HMGB1 occur in pathological foci of different types of epilepsy (76, 77). Glial activation plays an important role in the development of epilepsy, and HMGB1 may mediate microglial activation during epileptic seizures through the TLR4/NF-κB signaling pathway (78). HMGB1 activates the IL-1R/TLR signaling pathway in neurons and plays a key role in seizures and relapse by catalyzing the phosphorylation of the NR2B subunit of the *N*-methyl-D-aspartate (NMDA-NR2B) receptor *via* rapid sarcoma family kinases (79). HMGB1 affects neuronal excitability by inhibiting astrocyte glutamate transporter to increase extracellular glutamate concentration (80). It has been reported that phosphorylation of the NMDA-NR2B receptor upon activation by HMGB1/RAGE/TLR4 signaling results in Ca^2+^ influx, which increases neuronal cell excitability, which in turn induces epileptogenesis (59). Increased RAGE expression may also lead to neuronal hyperexcitability associated with amyloid-β synthesis (81). Among neurotransmitter receptors, TNF-α induces a rapid increase in neuronal synaptic expression of the amino-3-hydroxy-5-methyl-4-isoxazole propionic acid receptor (AMPAR) and acts on AMPAR to promote neuronal excitability (82). Seizures lead to brain cell damage, leading to passive release of HMGB1, creating a vicious cycle.

There are currently limited data on HMGB1 inhibitors in animal models of epilepsy. GL was neuroprotective against lithium/pilocarpine-induced status epilepticus (SE) in rats and ameliorated pilocarpine-induced oxidative damage and inflammatory responses by inhibiting gliosis and downregulating pro-inflammatory factors, but showed no antiepileptic activity (82) (Table 5). Anti-HMGB1 mAb may exert an antiepileptic effect by inhibiting the HMGB1-TLR4 regulatory axis, reducing seizure frequency (83) (Table 5). RAGE may play a dual role in epilepsy: Constitutively expressed neuronal RAGE contributes to hippocampal cornu ammonis (CA)1 cell survival early in SE and is detrimental in subsequent stages of epileptogenesis (before spontaneous seizures or after the first seizure), leading to increased neuronal excitability (77). Current studies have shown that HMGB1 inhibitors (GL and anti-HMGB1 mAb) can reduce the frequency of different types of seizures, but data are limited (86). In addition, short-term seizure remission does not predict its long-term prognosis.

**Table 5:** Studies targeting HMGB1 in epilepsy.

**S.N**.	**Study model**	**Treatment**	**Mode of inhibition**	**Observations**	**References**
1	Pilocarpine-induced SE mouse model	GL	Release inhibition	Decreases levels of malondialdehyde and glutathione in brain regions	(82)
2	Pilocarpine-induced epilepsy mouse model	anti-HMGB1 mAb	Neutralization	Attenuates damage such as increased intracellular space in the hippocampus caused by seizures in epileptic mice	(83)
3	Pentylenetetrazol-induced epilepsy zebrafish model	GL	Release inhibition	Anticonvulsant Inhibits HMGB1/TLR4/NF-κB signaling pathway	(84)
4	Lithium-pilocarpine induced epilepsy mouse model	GL	Release inhibition	Inhibits the translocation of HMGB1 from the nucleus to the cytoplasm Improves the neuronal damage in the CA1 and CA3 regions of the hippocampus after SE Protects BBB	(85)

## Autism

Autism is a type of neurodevelopmental disorder starting in early childhood, with characteristic symptoms of social interaction and communication disorder, and repetitive patterns of behavior. The pathogenesis of autism is currently unclear (87). From fetal development to adulthood, the immune system and the central nervous system interact with each other, and the activation of maternal immunity during fetal development can be a risk factor for autism. Patients with autism have altered immune responses, ranging from alterations in peripheral immune markers to increased activation of microglia in the central nervous system (CNS), all of which contribute to a chronic state of low-grade inflammation in the CNS (88). Clinical studies have found that plasma HMGB1 levels are elevated in ASD patients (89) (Table 6). Animal experiments have demonstrated that HMGB1 is associated with alterations in intestinal barrier function (93). At the same time, the serum HMGB1 level is positively correlated with the severity of autism, and the higher the HMGB1 level, the worse the social interaction ability (90). HMGB1 acts *via* HMGB1/RAGE/TLR4 axis, and activation of TLR4 signaling leads to the upregulation of NADPH oxidase 2 (NOX-2)-dependent reactive oxygen species (ROS) production by immune cells (94), increased vascular permeability, and leukocyte infiltration into nerve cells, resulting in persistent neuroinflammation (95). The neuropeptide oxytocin (OXT) can affect mood and social functioning and is therefore considered to be closely related to the pathophysiology of autism (96, 97). It was found that HMGB1 binds to endogenous secretory RAGE (esRAGE), resulting in a decrease in plasma RAGE levels, which in turn affects the transport of OXT from the periphery to the brain (14). Therefore, HMGB1 may be involved in the molecular pathway of immune dysfunction in individuals with ASD. Epidermal growth factor receptor (EGFR) is involved in the growth and differentiation of cells in the central nervous system, and studies have found that plasma EGFR levels are correlated with HMGB1. In addition, the study found that EGFR levels correlated with symptom severity in children with autism (98). However, there are few clinical and preclinical studies on autism at present, and more research is needed to clarify the role of HMGB1 in the pathophysiology of autism and to clarify the specific molecular mechanism by which HMGB1 is involved in autism.

**Table 6:** Overview of the original research studies investigating the role of HMGB1 in autism spectrum disorder.

**Authors**	**Study population**		**HMGB1 in ASD patients**		**Symptoms**
	**Patients**	**Controls**	**Serums**	**Fecal**	
Makris G (14)	42	38	↑	–	AQ attention to detail subscale SQ total score
Babinská K (89)	31	16	↑	–	GI sign severity
Emanuele E (90)	22	28	↑	–	ADI-R Social Scores
Russo AJ (91)	38	40	↑	–	–
Carissimi C (92)	30	14	–	↑	GI sign severity

## Depression

Depression manifests as a long-term physical and psychological downturn that affects ~300 million people worldwide (99). Stress is an indirect cause of depression, which induces depression-like behaviors through the HMGB1/TLR4/NF-κB signaling pathway in the hippocampus (100). Persistent expression of HMGB1/RAGE in microglia increases susceptibility to depression (101). The ventral medial prefrontal cortex (vmPFC) is one of the key brain regions involved in the pathogenesis of depression, and it plays a key role in the affective deficits of depression. Animal studies have found increased expression of inflammatory cytokines and decreased astrocytes in rats exposed to chronic unpredictable mild stress (CUMS) in vmPFC (102). The reduction of astrocytes in the prefrontal cortex (PFC) is considered to be one of the pathophysiological changes in depression (103). Chronic unpredictable mild stress (CUMS) induces nucleocytoplasmic translocation of HMGB1 in microglia and neurons (104). HMGB1 may lead to microglial activation and neuroinflammation through TLR4/NF-κB and TNF-α/TNFR1/NF-κB signaling pathways. This neuroinflammation-induced behavioral change is thought to be related to the activation of indoleamine-pyrrole 2,3-dioxygenase (IDO) in the kynurenine pathway and changes in neurotransmitter metabolism (5-hydroxytryptamine, 5-HT) (5). HMGB1 can activate the tryptophan degradation (canine purine) pathway and increase the activity of the rate-limiting enzyme IDO (105, 106). On the one hand, IDO catalyzes the conversion of tryptophan into neurotoxicity. Metabolites, such as quinolinic acid (QUIN), selectively bind to NMDAR, resulting in glutamate signaling and neuronal Ca influx, ultimately leading to excitotoxicity. At the same time, it also activates the secretion of glutamate in neurons (107). Both high concentrations of glutamate and QUIN enhance glutamatergic neurotransmission, leading to the development of depression (108); on the other hand, 5-HT biosynthesis is reduced and leads to depressive mood (109). Another possible mechanism of HMGB1-mediated depression involves damage to dopaminergic neurons. After exposure to stress, microglia secrete reactive oxygen species (ROS) and nitrogen (NOS), which may rapidly reduce the availability of neopterin and tetrahydrobiopterin (BH4), which in turn leads to the DA synthesis rate-limiting enzyme amphetamine amino acid hydroxylase (PAH) and tyrosine hydroxylase (TH) are inactivated and DA synthesis is blocked (110).

The HMGB1 inhibitors GL and EP can improve depression-like behaviors (104, 108). Arctigenin exhibits significant antidepressant effects in rodent models of depression, attenuates microglial activation and neuroinflammation through HMGB1/TLR4/NF-κB and TNF-α/TNFR1/NF-κB signaling pathways, and inhibits IDO increase and decrease in 5-HT (111). Minocycline can inhibit CUMS-induced HMGB1 nucleocytoplasmic translocation in microglia and neurons and improve behavioral and cognitive deficits in CUMS-depressed mice (104). In addition, inhibition of phosphodiesterase-4 (PDE4) can exert antidepressant effects by inhibiting the HMGB1/RAGE signaling pathway (112). TAK-242 (TLR4 inhibitor) can significantly inhibit dsHMGB1, downregulation of hippocampal myelin basic protein and upregulation of hippocampal TNF-α protein, and improve depressive behavior in rodents (15). The glutamate receptor antagonist ketamine and the IDO inhibitor 1-methyltryptophan can also improve depressive symptoms in rodents (110). In conclusion, inhibition of HMGB1 release or inhibition of HMGB1/TLR4/RAGE signaling pathway by HMGB1 inhibitors is beneficial for the treatment of depression (Tables 7–9). However, most of the study results are based on animal experiments and lack the verification of clinical research results.

**Table 7:** Studies targeting HMGB1 in depression.

**S.N**.	**Study model**	**Treatment**	**Mode of inhibition**	**Observations**	**References**
1	CUMS-induced depression mouse model	GL	Release inhibition	Improves chronic stress-induced depression	(106)
2	CUMS-induced depression mouse model	EP	Release inhibition	Depressed behavioral tendency	(108)
3	LPS-induced depression mouse model	GL	Release inhibition	Eliminates LPS-induced cognitive dysfunction, anxiety and depression-like behaviors	(113)
4	PSNL-induced depression mouse model	Anti-HMGB1 mAb	Neutralization	Reduces microglia activation and anxiety-depression-like behavior	(114)

**Table 8:** Studies targeting HMGB1/TLR4 pathway in depression.

**S.N**.	**Study model**	**Treatment**	**Mechanism**	**Observations**	**References**
1	CUMS-induced depression mouse model	BA	Inhibition of HMGB1/TLR4/NF-κB pathway	Inhibits HMGB1/TLR4/NF-κB pathway	(100)
2	CUMS-induced depression mouse model	AG	Inhibition of HMGB1/TLR4/NF-κB pathway	Inhibits HMGB1/TLR4/NF-κB pathway	(111)
3	LPS-induced depression mouse model	polydatin	Inhibition of HMGB1/NF-κB pathway	Inhibits Sirt1/HMGB1/NF-κB pathway	(115)

**Table 9:** Clinical studies targeting HMGB1 in depression.

**S.N**.	**Type of study**	**Treatment**		**Observations**	**limitation**	**References**
		**Experimental**	**Control**			
1	Randomized, double-blind, placebo-controlled clinical trial	SSRI + GL	SSRI + PBO	The SSRI + GL group had more relief of depressive symptoms than the SSRI+PBO group	The sample size of this study was not large enough and the follow-up time was relatively short	(116)

## Multiple sclerosis

Multiple sclerosis (MS) is an immune-mediated chronic inflammatory demyelinating disease of the central nervous system, often involving the brain, spinal cord, and optic nerves. At the same time, clinical studies have found that the concentration of HMGB1 in the serum and cerebrospinal fluid of MS patients is significantly increased (117). Experimental autoimmune encephalomyelitis (EAE) provides the most widely used MS experimental model (118). EAE model studies have found that HMGB1 may be released by activated macrophages and microglia during MS and induce neuroinflammation (119). Acetylated HMGB1 may be released during chronic inflammation in the clinically stable phase of MS, whereas HMGB1 may be in an unacetylated form during clinically relapsing acute inflammation (120). Serum HMGB1 levels can serve as a potential marker of MS activity and correlate with clinical relapse rates and disease duration (121). HMGB1 may be involved in the pathogenesis of MS by promoting autophagy. HMGB1 can further promote the binding of autophagy factor Beclin1 to type III phosphoinositide 3 kinase (PI3K Class III), thereby promoting the nucleation process of *ex vivo* membranes, thereby initiating autophagy (117). HMGB1 elevates inducible nitric oxide synthase (iNOS) and superoxide, leading to peroxynitrite (ONOO^−^) formation and increased pro-inflammatory factors (122). ONOO induces ceramide production in astrocytes, which in turn leads to demyelination, inhibition of remyelination, and increased BBB permeability. On the contrary, high levels of ceramides can promote cell death (122). In microglia, ceramides promote the assembly of NOD-like receptor pyrin domain containing 3 (NLRP3) inflammasome activation, thereby increasing the release of the IL-1β and IL-18, which further contributes to neuroinflammation (123). Furthermore, iNOS-mediated cytokine-induced nitric oxide excess can cause tissue damage in the central nervous system of EAE (124). Cellular senescence is a cellular feature of MS progenitor cells, and senescent neural progenitor cells can secrete HMGB1 oligodendrocyte progenitors (OPCs) to mature into myelinating oligodendrocytes (OLs), promoting chronic demyelination (125, 126).

HMGB1 monoclonal antibody has been shown to improve the progression of EAE (127) (Table 10). Meanwhile, HMGB1 promotes the release of Sonic hedgehog (Shh) through the HMGB1-RAGE signaling pathway, which can repair the BBB and reduce BBB permeability to promote axonal growth in spinal cord injury (129). Genetic inhibition of acid sphingomyelinase (aSMase)/ceramide prevents classic MS-like pathophysiology, including BBB disruption, leukocyte extravasation, and demyelination, in a model of EAE (130). Matrine (MAT) and GL alleviate inflammatory demyelination and activation of astrocytes and microglia/macrophages in the central nervous system of EAE rats by inhibiting HMGB1 (131, 132) (Tables 10, 11). The cumulative effect of HMGB1 will determine the outcome of the local inflammatory response of HMGB1 in terms of tissue damage. Blocking the HMGB1-RAGE interaction in damaged nerves reduces neurite outgrowth. On the contrary, inhibition of HMGB1 at inappropriate times may prevent tissue repair due to its role in neurite outgrowth and stem cell chemotaxis. Therefore, the role of HMGB1 in MS still needs further study.

**Table 10:** Studies targeting HMGB1 in MS.

**S.N**.	**Study model**	**Treatment**	**Mode of inhibition**	**Observations**	**References**
1	MOG-induced EAE mouse model	anti-HMGB1 mAb	Neutralization	Improves clinical and pathological severity of EAE	(127)
2	MOG-induced EAE mouse model	GL	Release inhibition	Decreases HMGB1 release	(128)

**Table 11:** Studies targeting HMGB1/TLR4 pathway in MS.

**S.N**.	**Study model**	**Treatment**	**Mechanism**	**Observations**	**References**
1	MOG-induced EAE mouse model	MAT	Inhibition of HMGB1/TLR4/NF-κB pathway	Relief of inflammatory demyelination and activation of astrocytes and microglia/macrophages in the central nervous system of EAE rats	(131)

## Amyotrophic lateral sclerosis

Amyotrophic lateral sclerosis (ALS) is a neurodegenerative disease that selectively damages motor neurons, resulting in rapid muscle wasting and weakness after onset (133). The pathogenesis of ALS is still unclear, but current studies have found that immune and inflammatory factors may be involved in the pathophysiology of ALS. HMGB1 induces neuroinflammation through the HMGB1/RAGE or HMGB1/TLR4 signaling pathway leading to increased release of tumor necrosis factor-α and interleukin (134). Serum levels of HMGB1 autoantibodies were upregulated in ALS patients compared with age-matched healthy controls (135). At the same time, the nucleocytoplasmic translocation of HMGB1 in reactive astrocytes and microglia was observed in ALS patients and mouse models (134). Therefore, HMGB1 may serve as a biomarker for ALS diagnosis and clinical assessment. In SOD1G93A mice exhibiting overt disease symptoms, HMGB1-immunopositive motor neurons progressively decreased, possibly due to passive release from damaged cells, whereas the subcellular distribution of HMGB1 in glial cells did not change, which helps stability and regulation of transcriptional activity during maintenance of its responsive response to motor neuron degeneration (136). The binding of HMGB1 to RAGE and TLR4 leads to the activation of NF-κB and inflammatory cytokines, the latter of which have been implicated in the pathogenesis of ALS. Animal studies have found that TLR4 signaling may lead to motor nerve death and, ultimately, ALS disease progression. Loss of TLR4 and RAGE can prolong survival and improve hindlimb grip strength (137, 138).

HMGB1 antibody improved early symptoms in SOD1G93A transgenic mice, but did not prolong survival or improve exercise performance (134) (Table 12). HMGB1 blockade therapy has limited efficacy in the SOD1G93A mouse model, possibly due to the presence of other endogenous ligands that activate TLR2, TLR4, and RAGE (134). On the contrary, astrocyte HMGB1 signaling in ALS can protect nerves by releasing neurotrophic factors, such as brain-derived neurotrophic factor and glial cell-derived neurotrophic factor (139).

**Table 12:** Studies targeting HMGB1 in ALS.

**S.N**.	**Study model**	**Treatment**	**Mode of inhibition**	**Observations**	**References**
1	Transgenic SOD1G93A mice	Anti-HMGB1 mAb	Neutralization	Briefly improves hindlimb grip strength in mice early in the disease, but not prolonged survival Reduces spinal cord TNF-α and complement C5a receptor 1 gene expression, but did not affect overall glial activation	(134)

## Conclusion

Neuroinflammation is thought to be involved in the pathogenesis of Parkinson's disease, stroke, traumatic brain injury, epilepsy, autism, depression, multiple sclerosis, and amyotrophic lateral sclerosis, and HMGB1 plays an important role as a neuroinflammatory mediator in the above diseases. Meanwhile, HMGB1 has the potential as a common biomarker for the aforementioned neurological diseases and may be an important therapeutic target for these neurological diseases. Anti-HMGB1 monoclonal antibodies and HMGB1 inhibitors have been shown to improve neurological symptoms in animal models of the above diseases within a specific therapeutic time window, providing a new therapeutic idea. Antagonists such as anti-HMGB1 monoclonal antibodies, ethyl pyruvate, inhibit HMGB1 by interfering with its cytoplasmic export, while other antagonists such as glycyrrhizin directly bind to HMGB1 and render its receptors unavailable. However, the current research still has certain limitations. Although clinical and preclinical studies have shown elevated levels of HMGB1 in the blood and cerebrospinal fluid of patients with Parkinson's disease, stroke, traumatic brain injury, epilepsy, autism, depression, multiple sclerosis, and amyotrophic lateral sclerosis, it is unclear to what extent HMGB1 contributes to the disease phenotype. In addition, most clinical or preclinical studies detect serum or cerebrospinal fluid HMGB1 content by ELISA, which cannot distinguish between active release of HMGB1 from immune cells or passive release from necrotic cells and cannot distinguish HMGB1 subtypes. Different isoforms of HMGB1 play different roles in the process of inflammation, and the currently studied HMGB1 inhibitors cannot target the harmful HMGB1 isoforms. Meanwhile, the duration of HMGB1 neutralization/inhibition by HMGB1 antagonists still needs further study. Although HMGB1 antagonists have yielded positive results in animal studies, clinical findings are limited. Finally, HMGB1 is thought to promote post-injury inflammation in vertebrates, but its benefit in neuroregeneration cannot be ruled out. Therefore, the role of HMGB1 in the nervous system injury response, the release mechanism of HMGB1, and the structure–function interaction with inflammatory receptors and downstream signaling pathways need to be further studied, and the clinical translation of HMGB1 antagonists still needs a lot of clinical research.

## Author contributions

DM, YZ, FX, and XH participated in writing the manuscript. HZ was responsible for critical reading of the manuscript. All authors read and approved the final version of manuscript.

## Conflict of interest

The authors declare that the research was conducted in the absence of any commercial or financial relationships that could be construed as a potential conflict of interest.

## Publisher's note

All claims expressed in this article are solely those of the authors and do not necessarily represent those of their affiliated organizations, or those of the publisher, the editors and the reviewers. Any product that may be evaluated in this article, or claim that may be made by its manufacturer, is not guaranteed or endorsed by the publisher.

## Abbreviations

OMT: oxymatrine
ROT: rotenone
ALO: alogliptin
MACO: middle cerebral artery occlusion
SSA: Saikosaponin A
Hp: haptoglobin
AG: Arctigenin
CCI: controlled cortical impact
PSNL: partial sciatic nerve ligation
BA: Baicalin
LPS: lipopolysaccharide
SSRI: selective serotonin reuptake inhibitor
PBO: placebo
MOG: myelin oligodendrocyte glycoprotein
AQ: autism spectrum quotient
SQ: systemizing quotient
GI score: gastrointestinal dysfunction scores
ADI-R: Autism Diagnostic Interview-Revised.

## References

[1] Goodwin GH Sanders C Johns EW. A new group of chromatin-associated proteins with a high content of acidic and basic amino acids. Eur J Biochem. (1973) 38:14–9. 10.1111/j.1432-1033.1973.tb03026.x 4774120

[2] Wang H Bloom O Zhang M Vishnubhakat JM Ombrellino M Che J. HMG-1 as a late mediator of endotoxin lethality in mice. Science. (1999) 285:248–51. 10.1126/science.285.5425.248 10398600

[3] Bustin M. Revised nomenclature for high mobility group (HMG) chromosomal proteins. Trends Biochem Sci. (2001) 26:152–3. 10.1016/S0968-0004(00)01777-1 11246012

[4] Souery D Papakostas GI Trivedi MH. Treatment-resistant depression. J Clin Psychiatry. (2006) 67(Suppl 6):16−22. 16848672

[5] Rana T Behl T Mehta V Uddin MS Bungau S. Molecular insights into the therapeutic promise of targeting HMGB1 in depression. Pharmacol Rep. (2021) 73:31–42. 10.1007/s43440-020-00163-6 33015736

[6] Xue J Suarez JS Minaai M Li S Gaudino G Pass HI. HMGB1 as a therapeutic target in disease. J Cell Physiol. (2021) 236:3406–19. 10.1002/jcp.30125 33107103 PMC8104204

[7] Frank MG Weber MD Watkins LR Maier SF. Stress sounds the alarmin: the role of the danger-associated molecular pattern HMGB1 in stress-induced neuroinflammatory priming. Brain Behav Immun. (2015) 48:1–7. 10.1016/j.bbi.2015.03.010 25816800 PMC4508196

[8] Singh V Roth S Veltkamp R Liesz A. HMGB1 as a key mediator of immune mechanisms in ischemic stroke. Antioxid Redox Signal. (2016) 24:635–51. 10.1089/ars.2015.6397 26493086

[9] Qu Y Zhan Y Yang S Ren S Qiu X Rehamn ZU. Newcastle disease virus infection triggers HMGB1 release to promote the inflammatory response. Virology. (2018) 525:19–31. 10.1016/j.virol.2018.09.001 30216776

[10] Baran A Bulut M Kaya MC Demirpençe Ö Sevim B Akil E. High-sensitivity C-reactive protein and high mobility group box-1 levels in Parkinson's disease. Neurol Sci. (2019) 40:167–73. 10.1007/s10072-018-3611-z 30353300

[11] Le K Mo S Lu X Idriss Ali A Yu D Guo Y. Association of circulating blood HMGB1 levels with ischemic stroke: a systematic review and meta-analysis. Neurol Res. (2018) 40:907–16. 10.1080/01616412.2018.1497254 30015578

[12] Webster KM Shultz SR Ozturk E Dill LK Sun M Casillas-Espinosa P. Targeting high-mobility group box protein 1 (HMGB1) in pediatric traumatic brain injury: chronic neuroinflammatory, behavioral, and epileptogenic consequences. Exp Neurol. (2019) 320:112979. 10.1016/j.expneurol.2019.112979 31229637

[13] Huang Q Liu J Shi Z Zhu X. Correlation of MMP-9 and HMGB1 expression with the cognitive function in patients with epilepsy and factors affecting the prognosis. Cell Mol Biol. (2020) 66:39–47. 10.14715/cmb/2020.66.3.6 32538745

[14] Makris G Chouliaras G Apostolakou F Papageorgiou C Chrousos GP Papassotiriou I. Increased serum concentrations of high mobility group box 1 (HMGB1) protein in children with autism spectrum disorder. Children. (2021) 8:478. 10.3390/children8060478 34198762 PMC8228126

[15] Lian YJ Gong H Wu TY Su WJ Zhang Y Yang YY. Ds-HMGB1 and fr-HMGB induce depressive behavior through neuroinflammation in contrast to nonoxid-HMGB1. Brain Behav Immun. (2017) 59:322–32. 10.1016/j.bbi.2016.09.017 27647532

[16] Bucova M Majernikova B Durmanova V Cudrakova D Gmitterova K Lisa I. HMGB1 as a potential new marker of disease activity in patients with multiple sclerosis. Neurol Sci. (2020) 41:599–604. 10.1007/s10072-019-04136-3 31728855

[17] Hwang CS Liu GT Chang MD Liao IL Chang HT. Elevated serum autoantibody against high mobility group box 1 as a potent surrogate biomarker for amyotrophic lateral sclerosis. Neurobiol Dis. (2013) 58:13–8. 10.1016/j.nbd.2013.04.013 23639787

[18] Chatterjee M van Steenoven I Huisman E Oosterveld L Berendse H van der Flier WM. Contactin-1 is reduced in cerebrospinal fluid of Parkinson's disease patients and is present within lewy bodies. Biomolecules. (2020) 10:1177. 10.3390/biom10081177 32806791 PMC7463939

[19] Novellino F Saccà V Donato A Zaffino P Spadea MF Vismara M. Innate immunity: a common denominator between neurodegenerative and neuropsychiatric diseases. Int J Mol Sci. (2020) 21:1115. 10.3390/ijms21031115 32046139 PMC7036760

[20] Santoro M Maetzler W Stathakos P Martin HL Hobert MA Rattay TW. *In-vivo* evidence that high mobility group box 1 exerts deleterious effects in the 1-methyl-4-phenyl-1,2,3,6-tetrahydropyridine model and Parkinson's disease which can be attenuated by glycyrrhizin. Neurobiol Dis. (2016) 91:59–68. 10.1016/j.nbd.2016.02.018 26921471 PMC4867789

[21] Lindersson EK Højrup P Gai WP Locker D Martin D Jensen PH. alpha-Synuclein filaments bind the transcriptional regulator HMGB-1. Neuroreport. (2004) 15:2735−9. 15597044

[22] Harms AS Thome AD Yan Z Schonhoff AM Williams GP Li X. Peripheral monocyte entry is required for alpha-Synuclein induced inflammation and neurodegeneration in a model of Parkinson disease. Exp Neurol. (2018) 300:179–87. 10.1016/j.expneurol.2017.11.010 29155051 PMC5759972

[23] Gao HM Zhou H Zhang F Wilson BC Kam W Hong JS. HMGB1 acts on microglia Mac1 to mediate chronic neuroinflammation that drives progressive neurodegeneration. J Neurosci. (2011) 31:1081–92. 10.1523/JNEUROSCI.3732-10.2011 21248133 PMC3046932

[24] Tang D Kang R Livesey KM Cheh CW Farkas A Loughran P. Endogenous HMGB1 regulates autophagy. J Cell Biol. (2010) 190:881–92. 10.1083/jcb.200911078 20819940 PMC2935581

[25] Karim MR Liao EE Kim J Meints J Martinez HM Pletnikova O. α-Synucleinopathy associated c-Abl activation causes p53-dependent autophagy impairment. Mol Neurodegener. (2020) 15:27. 10.1186/s13024-020-00364-w 32299471 PMC7164361

[26] Wang K Huang J Xie W Huang L Zhong C Chen Z. Beclin1 and HMGB1 ameliorate the α-synuclein-mediated autophagy inhibition in PC12 cells. Diagn Pathol. (2016) 11:15. 10.1186/s13000-016-0459-5 26822891 PMC4731928

[27] Kim SJ Ryu MJ Han J Jang Y Lee MJ Ju X. Non-cell autonomous modulation of tyrosine hydroxylase by HMGB1 released from astrocytes in an acute MPTP-induced Parkinsonian mouse model. Lab Invest. (2019) 99:1389–99. 10.1038/s41374-019-0254-5 31043679

[28] Sasaki T Liu K Agari T Yasuhara T Morimoto J Okazaki M. Anti-high mobility group box 1 antibody exerts neuroprotection in a rat model of Parkinson's disease. Exp Neurol. (2016) 275(Pt 1):220–31. 10.1016/j.expneurol.2015.11.003 26555088

[29] Tian Y Cao Y Chen R Jing Y Xia L Zhang S. HMGB1 A box protects neurons by potently inhibiting both microglia and T cell-mediated inflammation in a mouse Parkinson's disease model. Clin Sci. (2020) 134:2075–90. 10.1042/CS20200553 32706028

[30] Ren Q Jiang X Paudel YN Gao X Gao D Zhang P. Co-treatment with natural HMGB1 inhibitor Glycyrrhizin exerts neuroprotection and reverses Parkinson's disease like pathology in Zebrafish. J Ethnopharmacol. (2022) 292:115234. 10.1016/j.jep.2022.115234 35358621

[31] Gan P Ding L Hang G Xia Q Huang Z Ye X. Oxymatrine attenuates dopaminergic neuronal damage and microglia-mediated neuroinflammation through cathepsin D-dependent HMGB1/TLR4/NF-κB pathway in Parkinson's disease. Front Pharmacol. (2020) 11:776. 10.3389/fphar.2020.00776 32528295 PMC7264119

[32] Safar MM Abdelkader NF Ramadan E Kortam MA Mohamed AF. Novel mechanistic insights towards the repositioning of alogliptin in Parkinson's disease. Life Sci. (2021) 287:120132. 10.1016/j.lfs.2021.120132 34774622

[33] Zhang K Tu M Gao W Cai X Song F Chen Z. Hollow Prussian blue nanozymes drive neuroprotection against ischemic stroke *via* attenuating oxidative stress, counteracting inflammation, and suppressing cell apoptosis. Nano Lett. (2019) 19:2812–23. 10.1021/acs.nanolett.8b04729 30908916

[34] Nazari S Rameshrad M Hosseinzadeh H. Toxicological effects of *Glycyrrhiza glabra* (Licorice): a review. Phytother Res. (2017) 31:1635–50. 10.1002/ptr.5893 28833680

[35] Seo MS Kim HJ Kim H Park SW. Ethyl pyruvate directly attenuates active secretion of HMGB1 in proximal tubular cells *via* induction of heme oxygenase-1. J Clin Med. (2019) 8:629. 10.3390/jcm8050629 31072024 PMC6572201

[36] Woodruff TM Thundyil J Tang SC Sobey CG Taylor SM Arumugam TV. Pathophysiology, treatment, and animal and cellular models of human ischemic stroke. Mol Neurodegener. (2011) 6:11. 10.1186/1750-1326-6-11 21266064 PMC3037909

[37] Zhang J Takahashi HK Liu K Wake H Liu R Maruo T. Anti-high mobility group box-1 monoclonal antibody protects the blood-brain barrier from ischemia-induced disruption in rats. Stroke. (2011) 42:1420–8. 10.1161/STROKEAHA.110.598334 21474801

[38] Richard SA Sackey M Su Z Xu H. Pivotal neuroinflammatory and therapeutic role of high mobility group box 1 in ischemic stroke. Biosci Rep. (2017) 37:BSR20171104. 10.1042/BSR20171104 29054968 PMC5715129

[39] Kim JB Sig Choi J Yu YM Nam K Piao CS Kim S. HMGB1, a novel cytokine-like mediator linking acute neuronal death and delayed neuroinflammation in the postischemic brain. J Neurosci. (2006) 26:6413–21. 10.1523/JNEUROSCI.3815-05.2006 16775128 PMC6674036

[40] Nishibori M Wang D Ousaka D Wake H. High mobility group box-1 and blood-brain barrier disruption. Cells. (2020) 9:2650. 10.3390/cells9122650 33321691 PMC7764171

[41] Qiu J Nishimura M Wang Y Sims JR Qiu S Savitz SI. Early release of HMGB-1 from neurons after the onset of brain ischemia. J Cereb Blood Flow Metab. (2008) 28:927–38. 10.1038/sj.jcbfm.9600582 18000511

[42] Lei C Lin S Zhang C Tao W Dong W Hao Z. High-mobility group box1 protein promotes neuroinflammation after intracerebral hemorrhage in rats. Neuroscience. (2013) 228:190–9. 10.1016/j.neuroscience.2012.10.023 23085216

[43] Pedrazzi M Raiteri L Bonanno G Patrone M Ledda S Passalacqua M. Stimulation of excitatory amino acid release from adult mouse brain glia subcellular particles by high mobility group box 1 protein. J Neurochem. (2006) 99:827–38. 10.1111/j.1471-4159.2006.04120.x 16911580

[44] Kikuchi K Kawahara K Biswas KK Ito T Tancharoen S Morimoto Y. Minocycline attenuates both OGD-induced HMGB1 release and HMGB1-induced cell death in ischemic neuronal injury in PC12 cells. Biochem Biophys Res Commun. (2009) 385:132–6. 10.1016/j.bbrc.2009.04.041 19379716

[45] Kikuchi K Kawahara K Tancharoen S Matsuda F Morimoto Y Ito T. The free radical scavenger edaravone rescues rats from cerebral infarction by attenuating the release of high-mobility group box-1 in neuronal cells. J Pharmacol Exp Ther. (2009) 329:865–74. 10.1124/jpet.108.149484 19293391

[46] Kim ID Lee H Kim SW Lee HK Choi J Han PL. Alarmin HMGB1 induces systemic and brain inflammatory exacerbation in post-stroke infection rat model. Cell Death Dis. (2018) 9:426. 10.1038/s41419-018-0438-8 29555931 PMC5859283

[47] Liu K Mori S Takahashi HK Tomono Y Wake H Kanke T. Anti-high mobility group box 1 monoclonal antibody ameliorates brain infarction induced by transient ischemia in rats. FASEB J. (2007) 21:3904–16. 10.1096/fj.07-8770com 17628015

[48] Chen H Guan B Wang B Pu H Bai X Chen X. Glycyrrhizin prevents hemorrhagic transformation and improves neurological outcome in ischemic stroke with delayed thrombolysis through targeting peroxynitrite-mediated HMGB1 signaling. Transl Stroke Res. (2020) 11:967–82. 10.1007/s12975-019-00772-1 31872339

[49] Nishibori M Mori S Takahashi HK. Anti-HMGB1 monoclonal antibody therapy for a wide range of CNS and PNS diseases. J Pharmacol Sci. (2019) 140:94–101. 10.1016/j.jphs.2019.04.006 31105025

[50] Lei C Zhang S Cao T Tao W Liu M Wu B. Corrigendum to “HMGB1 may act *via* RAGE to promote angiogenesis in the later phase after intracerebral hemorrhage” [Neuroscience 295 (2015) 39-47]. Neuroscience. (2022) 481:238–9. 10.1016/j.neuroscience.2021.11.041 34906390

[51] Tian X Liu C Shu Z Chen G. Review: therapeutic targeting of HMGB1 in stroke. Curr Drug Deliv. (2017) 14:785–90. 10.2174/1567201813666160808111933 27501713

[52] Zhu JR Lu HD Guo C Fang WR Zhao HD Zhou JS. Berberine attenuates ischemia-reperfusion injury through inhibiting HMGB1 release and NF-κB nuclear translocation. Acta Pharmacol Sin. (2018) 39:1706–15. 10.1038/s41401-018-0160-1 30266998 PMC6289370

[53] Wang X Yang G. Saikosaponin A attenuates neural injury caused by ischemia/reperfusion. Transl Neurosci. (2020) 11:227–35. 10.1515/tnsci-2020-0129 33335763 PMC7712316

[54] Morimoto M Nakano T Egashira S Irie K Matsuyama K Wada M. Haptoglobin regulates macrophage/microglia-induced inflammation and prevents ischemic brain damage *via* binding to HMGB1. J Am Heart Assoc. (2022) 11:e024424. 10.1161/JAHA.121.024424 35243897 PMC9075294

[55] Halder SK Ueda H. Amlexanox inhibits cerebral ischemia-induced delayed astrocytic high-mobility group box 1 release and subsequent brain damage. J Pharmacol Exp Ther. (2018) 365:27–36. 10.1124/jpet.117.245340 29330155

[56] Maas A Menon DK Adelson PD Andelic N Bell MJ Belli A. Traumatic brain injury: integrated approaches to improve prevention, clinical care, and research. Lancet Neurol. (2017) 16:987–1048. 10.1016/S1474-4422(17)30371-X 29122524

[57] Wang KY Yu GF Zhang ZY Huang Q Dong XQ. Plasma high-mobility group box 1 levels and prediction of outcome in patients with traumatic brain injury. Clin Chim Acta. (2012) 413:1737–41. 10.1016/j.cca.2012.07.002 22789964

[58] Gao TL Yuan XT Yang D Dai HL Wang WJ Peng X. Expression of HMGB1 and RAGE in rat and human brains after traumatic brain injury. J Trauma Acute Care Surg. (2012) 72:643–9. 10.1097/TA.0b013e31823c54a6 22491548

[59] Manivannan S Harari B Muzaffar M Elalfy O Hettipathirannahelage S James Z. Glycyrrhizin blocks the detrimental effects of HMGB1 on cortical neurogenesis after traumatic neuronal injury. Brain Sci. (2020) 10:760. 10.3390/brainsci10100760 33096930 PMC7593920

[60] Webster KM Sun M Crack PJ O'Brien TJ Shultz SR Semple BD. Age-dependent release of high-mobility group box protein-1 and cellular neuroinflammation after traumatic brain injury in mice. J Comp Neurol. (2019) 527:1102–17. 10.1002/cne.24589 30499129

[61] Au AK Aneja RK Bell MJ Bayir H Feldman K Adelson PD. Cerebrospinal fluid levels of high-mobility group box 1 and cytochrome C predict outcome after pediatric traumatic brain injury. J Neurotrauma. (2012) 29:2013–21. 10.1089/neu.2011.2171 22540160 PMC3408241

[62] Pang H Huang T Song J Li D Zhao Y Ma X. Inhibiting HMGB1 with glycyrrhizic acid protects brain injury after DAI *via* its anti-inflammatory effect. Mediators Inflamm. (2016) 2016:4569521. 10.1155/2016/4569521 27041825 PMC4799817

[63] Su X Wang H Zhao J Pan H Mao L. Beneficial effects of ethyl pyruvate through inhibiting high-mobility group box 1 expression and TLR4/NF-κB pathway after traumatic brain injury in the rat. Mediators Inflamm. (2011) 2011:807142. 10.1155/2011/807142 21772666 PMC3136093

[64] Tan SW Zhao Y Li P Ning YL Huang ZZ Yang N. HMGB1 mediates cognitive impairment caused by the NLRP3 inflammasome in the late stage of traumatic brain injury. J Neuroinflammation. (2021) 18:241. 10.1186/s12974-021-02274-0 34666797 PMC8527642

[65] Paudel YN Angelopoulou E Piperi C Othman I Shaikh MF. HMGB1-mediated neuroinflammatory responses in brain injuries: potential mechanisms and therapeutic opportunities. Int J Mol Sci. (2020) 21:4609. 10.3390/ijms21134609 32610502 PMC7370155

[66] Gao T Chen Z Chen H Yuan H Wang Y Peng X. Inhibition of HMGB1 mediates neuroprotection of traumatic brain injury by modulating the microglia/macrophage polarization. Biochem Biophys Res Commun. (2018) 497:430–6. 10.1016/j.bbrc.2018.02.102 29448108

[67] Yang L Wang F Yang L Yuan Y Chen Y Zhang G. HMGB1 a-box reverses brain edema and deterioration of neurological function in a traumatic brain injury mouse model. Cell Physiol Biochem. (2018) 46:2532–42. 10.1159/000489659 29742510

[68] Chen X Chen C Fan S Wu S Yang F Fang Z. Omega-3 polyunsaturated fatty acid attenuates the inflammatory response by modulating microglia polarization through SIRT1-mediated deacetylation of the HMGB1/NF-κB pathway following experimental traumatic brain injury. J Neuroinflammation. (2018) 15:116. 10.1186/s12974-018-1151-3 29678169 PMC5909267

[69] Okuma Y Wake H Teshigawara K Takahashi Y Hishikawa T Yasuhara T. Anti-high mobility group box 1 antibody therapy may prevent cognitive dysfunction after traumatic brain injury. World Neurosurg. (2019) 122:e864–71. 10.1016/j.wneu.2018.10.164 30391757

[70] Chen X Wu S Chen C Xie B Fang Z Hu W. Omega-3 polyunsaturated fatty acid supplementation attenuates microglial-induced inflammation by inhibiting the HMGB1/TLR4/NF-κB pathway following experimental traumatic brain injury. J Neuroinflammation. (2017) 14:143. 10.1186/s12974-017-0917-3 28738820 PMC5525354

[71] Andersson U Yang H Harris H. High-mobility group box 1 protein (HMGB1) operates as an alarmin outside as well as inside cells. Semin Immunol. (2018) 38:40–8. 10.1016/j.smim.2018.02.011 29530410

[72] Riazi K Galic MA Pittman QJ. Contributions of peripheral inflammation to seizure susceptibility: cytokines and brain excitability. Epilepsy Res. (2010) 89:34–42. 10.1016/j.eplepsyres.2009.09.004 19804959

[73] Pauletti A Terrone G Shekh-Ahmad T Salamone A Ravizza T Rizzi M. Targeting oxidative stress improves disease outcomes in a rat model of acquired epilepsy. Brain. (2019) 142:e39. 10.1093/brain/awz130 31145451 PMC6598637

[74] Kan M Song L Zhang X Zhang J Fang P. Circulating high mobility group box-1 and toll-like receptor 4 expressions increase the risk and severity of epilepsy. Braz J Med Biol Res. (2019) 52:e7374. 10.1590/1414-431x20197374 31241711 PMC6596364

[75] Zhu M Chen J Guo H Ding L Zhang Y Xu Y. High mobility group protein B1 (HMGB1) and interleukin-1β as prognostic biomarkers of epilepsy in children. J Child Neurol. (2018) 33:909–17. 10.1177/0883073818801654 30303442

[76] Maroso M Balosso S Ravizza T Liu J Aronica E Iyer AM. Toll-like receptor 4 and high-mobility group box-1 are involved in ictogenesis and can be targeted to reduce seizures. Nat Med. (2010) 16:413–9. 10.1038/nm.2127 20348922

[77] Iori V Maroso M Rizzi M Iyer AM Vertemara R Carli M. Receptor for advanced glycation endproducts is upregulated in temporal lobe epilepsy and contributes to experimental seizures. Neurobiol Dis. (2013) 58:102–14. 10.1016/j.nbd.2013.03.006 23523633

[78] Shi Y Zhang L Teng J Miao W. HMGB1 mediates microglia activation *via* the TLR4/NF-κB pathway in coriaria lactone induced epilepsy. Mol Med Rep. (2018) 17:5125–31. 10.3892/mmr.2018.8485 29393419 PMC5865977

[79] Paudel YN Shaikh MF Chakraborti A Kumari Y Aledo-Serrano Á Aleksovska K. HMGB1: a common biomarker and potential target for TBI, neuroinflammation, epilepsy, and cognitive dysfunction. Front Neurosci. (2018) 12:628. 10.3389/fnins.2018.00628 30271319 PMC6142787

[80] Terrone G Balosso S Pauletti A Ravizza T Vezzani A. Inflammation and reactive oxygen species as disease modifiers in epilepsy. Neuropharmacology. (2020) 167:107742. 10.1016/j.neuropharm.2019.107742 31421074

[81] Scharfman HE. “Untangling” Alzheimer's disease and epilepsy. Epilepsy Curr. (2012) 12:178–83. 10.5698/1535-7511-12.5.178 23118602 PMC3482723

[82] González-Reyes S Santillán-Cigales JJ Jiménez-Osorio AS Pedraza-Chaverri J Guevara-Guzmán R. Glycyrrhizin ameliorates oxidative stress and inflammation in hippocampus and olfactory bulb in lithium/pilocarpine-induced status epilepticus in rats. Epilepsy Res. (2016) 126:126–33. 10.1016/j.eplepsyres.2016.07.007 27490898

[83] Ying C Ying L Yanxia L Le W Lili C. High mobility group box 1 antibody represses autophagy and alleviates hippocampus damage in pilocarpine-induced mouse epilepsy model. Acta Histochem. (2020) 122:151485. 10.1016/j.acthis.2019.151485 31870503

[84] Paudel YN Khan SU Othman I Shaikh MF. Naturally occurring HMGB1 inhibitor, glycyrrhizin, modulates chronic seizures-induced memory dysfunction in zebrafish model. ACS Chem Neurosci. (2021) 12:3288–302. 10.1021/acschemneuro.0c00825 34463468

[85] Li YJ Wang L Zhang B Gao F Yang CM. Glycyrrhizin, an HMGB1 inhibitor, exhibits neuroprotective effects in rats after lithium-pilocarpine-induced status epilepticus. J Pharm Pharmacol. (2019) 71:390–9. 10.1111/jphp.13040 30417405

[86] Ravizza T Vezzani A. Pharmacological targeting of brain inflammation in epilepsy: therapeutic perspectives from experimental and clinical studies. Epilepsia Open. (2018) 3:133–42. 10.1002/epi4.12242 30564772 PMC6293065

[87] Dipasquale V Cutrupi MC Colavita L Manti S Cuppari C Salpietro C. Neuroinflammation in autism spectrum disorders: role of high mobility group box 1 protein. Int J Mol Cell Med. (2017) 6:148–55. 10.22088/acadpub.BUMS.6.3.148 29682486 PMC5898638

[88] Barbosa IG Rodrigues DH Rocha NP Sousa LF Vieira EL Simões-E-Silva AC. Plasma levels of alarmin IL-33 are unchanged in autism spectrum disorder: a preliminary study. J Neuroimmunol. (2015) 278:69–72. 10.1016/j.jneuroim.2014.11.021 25595254

[89] Babinská K Bucová M Durmanová V Lakatošová S Jánošíková D Bakoš J. Increased plasma levels of the high mobility group box 1 protein (HMGB1) are associated with a higher score of gastrointestinal dysfunction in individuals with autism. Physiol Res. (2014) 63:S613–8. 10.33549/physiolres.932932 25669692

[90] Emanuele E Boso M Brondino N Pietra S Barale F Ucelli di Nemi S. Increased serum levels of high mobility group box 1 protein in patients with autistic disorder. Prog Neuropsychopharmacol Biol Psychiatry. (2010) 34:681–3. 10.1016/j.pnpbp.2010.03.020 20302902

[91] Russo AJ. Decreased epidermal growth factor (EGF) associated with HMGB1 and increased hyperactivity in children with autism. Biomarker Insights. (2013) 8:35–41. 10.4137/BMI.S11270 23645980 PMC3623607

[92] Carissimi C Laudadio I Palone F Fulci V Cesi V Cardona F. Functional analysis of gut microbiota and immunoinflammation in children with autism spectrum disorders. Dig Liver Dis. (2019) 51:1366–74. 10.1016/j.dld.2019.06.006 31320306

[93] Sappington PL Yang R Yang H Tracey KJ Delude RL Fink MP. HMGB1 B box increases the permeability of Caco-2 enterocytic monolayers and impairs intestinal barrier function in mice. Gastroenterology. (2002) 123:790–802. 10.1053/gast.2002.35391 12198705

[94] Nadeem A Ahmad SF Bakheet SA Al-Harbi NO Al-Ayadhi LY Attia SM. Toll-like receptor 4 signaling is associated with upregulated NADPH oxidase expression in peripheral T cells of children with autism. Brain Behav Immun. (2017) 61:146–54. 10.1016/j.bbi.2016.12.024 28034626

[95] Nadeem A Siddiqui N Alharbi NO Alharbi MM. Airway and systemic oxidant-antioxidant dysregulation in asthma: a possible scenario of oxidants spill over from lung into blood. Pulm Pharmacol Ther. (2014) 29:31–40. 10.1016/j.pupt.2014.06.001 24929073

[96] Huang Y Huang X Ebstein RP Yu R. Intranasal oxytocin in the treatment of autism spectrum disorders: a multilevel meta-analysis. Neurosci Biobehav Rev. (2021) 122:18–27. 10.1016/j.neubiorev.2020.12.028 33400920

[97] Husarova VM Lakatosova S Pivovarciova A Babinska K Bakos J Durdiakova J. Plasma oxytocin in children with autism and its correlations with behavioral parameters in children and parents. Psychiatry Investig. (2016) 13:174–83. 10.4306/pi.2016.13.2.174 27081377 PMC4823192

[98] Russo AJ. Increased epidermal growth factor receptor (EGFR) associated with hepatocyte growth factor (HGF) and symptom severity in children with autism spectrum disorders (ASDs). J Cent Nerv Syst Dis. (2014) 6:79–83. 10.4137/JCNSD.S13767 25249767 PMC4167315

[99] Yang F Zhu W Cai X Zhang W Yu Z Li X. Minocycline alleviates NLRP3 inflammasome-dependent pyroptosis in monosodium glutamate-induced depressive rats. Biochem Biophys Res Commun. (2020) 526:553–9. 10.1016/j.bbrc.2020.02.149 32245616

[100] Liu L Dong Y Shan X Li L Xia B Wang H. Anti-depressive effectiveness of Baicalin *in vitro* and *in vivo*. Molecules. (2019) 24:326. 10.3390/molecules24020326 30658416 PMC6359445

[101] Franklin TC Wohleb ES Zhang Y Fogaça M Hare B Duman RS. Persistent increase in microglial RAGE contributes to chronic stress-induced priming of depressive-like behavior. Biol Psychiatry. (2018) 83:50–60. 10.1016/j.biopsych.2017.06.034 28882317 PMC6369917

[102] Fan C Song Q Wang P Li Y Yang M Yu SY. Neuroprotective EFFECTS of ginsenoside-Rg1 against depression-like behaviors *via* suppressing glial activation, synaptic deficits, and neuronal apoptosis in rats. Front Immunol. (2018) 9:2889. 10.3389/fimmu.2018.02889 30581440 PMC6292928

[103] Banasr M Duman RS. Glial loss in the prefrontal cortex is sufficient to induce depressive-like behaviors. Biol Psychiatry. (2008) 64:863–70. 10.1016/j.biopsych.2008.06.008 18639237 PMC2709733

[104] Wang B Huang X Pan X Zhang T Hou C Su WJ. Minocycline prevents the depressive-like behavior through inhibiting the release of HMGB1 from microglia and neurons. Brain Behav Immun. (2020) 88:132–43. 10.1016/j.bbi.2020.06.019 32553784

[105] Wang B Lian YJ Su WJ Liu LL Li JM Jiang CL. Fr-HMGB1 and ds-HMGB1 activate the kynurenine pathway *via* different mechanisms in association with depressive-like behavior. Mol Med Rep. (2019) 20:359–67. 10.3892/mmr.2019.10225 31115516 PMC6580048

[106] Wang B Lian YJ Dong X Peng W Liu LL Su WJ. Glycyrrhizic acid ameliorates the kynurenine pathway in association with its antidepressant effect. Behav Brain Res. (2018) 353:250–7. 10.1016/j.bbr.2018.01.024 29366745

[107] Maddison DC Giorgini F. The kynurenine pathway and neurodegenerative disease. Semin Cell Dev Biol. (2015) 40:134–41. 10.1016/j.semcdb.2015.03.002 25773161

[108] Wang B Lian YJ Su WJ Peng W Dong X Liu LL. HMGB1 mediates depressive behavior induced by chronic stress through activating the kynurenine pathway. Brain Behav Immun. (2018) 72:51–60. 10.1016/j.bbi.2017.11.017 29195782

[109] Myint AM Kim YK. Network beyond IDO in psychiatric disorders: revisiting neurodegeneration hypothesis. Prog Neuropsychopharmacol Biol Psychiatry. (2014) 48:304–13. 10.1016/j.pnpbp.2013.08.008 24184687

[110] Zhang H Ding L Shen T Peng D. HMGB1 involved in stress-induced depression and its neuroinflammatory priming role: a systematic review. Gen Psychiatry. (2019) 32:e100084. 10.1136/gpsych-2019-100084 31552388 PMC6738663

[111] Xu X Piao HN Aosai F Zeng XY Cheng JH Cui YX. Arctigenin protects against depression by inhibiting microglial activation and neuroinflammation *via* HMGB1/TLR4/NF-κB and TNF-α/TNFR1/NF-κB pathways. Br J Pharmacol. (2020) 177:5224–45. 10.1111/bph.15261 32964428 PMC7589024

[112] Xie J Bi B Qin Y Dong W Zhong J Li M. Inhibition of phosphodiesterase-4 suppresses HMGB1/RAGE signaling pathway and NLRP3 inflammasome activation in mice exposed to chronic unpredictable mild stress. Brain Behav Immun. (2021) 92:67–77. 10.1016/j.bbi.2020.11.029 33221489

[113] Ghosh D Singh A Kumar A Sinha N. High mobility group box 1 (HMGB1) inhibition attenuates lipopolysaccharide-induced cognitive dysfunction and sickness-like behavior in mice. Immunol Res. (2022). 10.1007/s12026-022-09295-8 35670903

[114] Hisaoka-Nakashima K Tomimura Y Yoshii T Ohata K Takada N Zhang FF. High-mobility group box 1-mediated microglial activation induces anxiodepressive-like behaviors in mice with neuropathic pain. Prog Neuropsychopharmacol Biol Psychiatry. (2019) 92:347–62. 10.1016/j.pnpbp.2019.02.005 30763674

[115] Bian H Xiao L Liang L Xie Y Wang H Slevin M. (2022). Polydatin prevents neuroinflammation and relieves depression *via* regulating Sirt1/HMGB1/NF-κB signaling in mice. Neurotox Res, 40, 1393–404. 10.1007/s12640-022-00553-z 35986876

[116] Cao ZY Liu YZ Li JM Ruan YM Yan WJ Zhong SY. Glycyrrhizic acid as an adjunctive treatment for depression through anti-inflammation: a randomized placebo-controlled clinical trial. J Affect Disord. (2020) 265:247–54. 10.1016/j.jad.2020.01.048 32090748

[117] Zhen C Wang Y Li D Zhang W Zhang H Yu X. Relationship of High-mobility group box 1 levels and multiple sclerosis: a systematic review and meta-analysis. Mult Scler Relat Disord. (2019) 31:87–92. 10.1016/j.msard.2019.03.030 30953953

[118] Constantinescu CS Farooqi N O'Brien K Gran B. Experimental autoimmune encephalomyelitis (EAE) as a model for multiple sclerosis (MS). Br J Pharmacol. (2011) 164:1079–106. 10.1111/j.1476-5381.2011.01302.x 21371012 PMC3229753

[119] Andersson A Covacu R Sunnemark D Danilov AI Dal Bianco A Khademi M. Pivotal advance: HMGB1 expression in active lesions of human and experimental multiple sclerosis. J Leukoc Biol. (2008) 84:1248–55. 10.1189/jlb.1207844 18644848

[120] Sternberg Z Sternberg D Chichelli T Drake A Patel N Kolb C. High-mobility group box 1 in multiple sclerosis. Immunol Res. (2016) 64:385–91. 10.1007/s12026-015-8673-x 26100980

[121] Malhotra S Fissolo N Tintoré M Wing AC Castilló J Vidal-Jordana A. Role of high mobility group box protein 1 (HMGB1) in peripheral blood from patients with multiple sclerosis. J Neuroinflammation. (2015) 12:48. 10.1186/s12974-015-0269-9 25879961 PMC4359557

[122] Anderson G Rodriguez M Reiter RJ. Multiple sclerosis: melatonin, orexin, and ceramide interact with platelet activation coagulation factors and gut-microbiome-derived butyrate in the circadian dysregulation of mitochondria in glia and immune cells. Int J Mol Sci. (2019) 20:5500. 10.3390/ijms20215500 31694154 PMC6862663

[123] Scheiblich H Schlütter A Golenbock DT Latz E Martinez-Martinez P Heneka MT. Activation of the NLRP3 inflammasome in microglia: the role of ceramide. J Neurochem. (2017) 143:534–50. 10.1111/jnc.14225 28940479

[124] Okuda Y Nakatsuji Y Fujimura H Esumi H Ogura T Yanagihara T. Expression of the inducible isoform of nitric oxide synthase in the central nervous system of mice correlates with the severity of actively induced experimental allergic encephalomyelitis. J Neuroimmunol. (1995) 62:103–12. 10.1016/0165-5728(95)00114-H 7499486

[125] Nicaise AM Wagstaff LJ Willis CM Paisie C Chandok H Robson P. Cellular senescence in progenitor cells contributes to diminished remyelination potential in progressive multiple sclerosis. Proc Natl Acad Sci U S A. (2019) 116:9030–9. 10.1073/pnas.1818348116 30910981 PMC6500153

[126] Rouillard ME Hu J Sutter PA Kim HW Huang JK Crocker SJ. The cellular senescence factor extracellular HMGB1 directly inhibits oligodendrocyte progenitor cell differentiation and impairs CNS remyelination. Front Cell Neurosci. (2022) 16:833186. 10.3389/fncel.2022.833186 35573828 PMC9095917

[127] Uzawa A Mori M Taniguchi J Masuda S Muto M Kuwabara S. Anti-high mobility group box 1 monoclonal antibody ameliorates experimental autoimmune encephalomyelitis. Clin Exp Immunol. (2013) 172:37–43. 10.1111/cei.12036 23480183 PMC3719929

[128] Sun Y Chen H Dai J Wan Z Xiong P Xu Y. Glycyrrhizin protects mice against experimental autoimmune encephalomyelitis by inhibiting high-mobility group box 1 (HMGB1) expression and neuronal HMGB1 release. Front Immunol. (2018) 9:1518. 10.3389/fimmu.2018.01518 30013568 PMC6036111

[129] Xiao Y Sun Y Liu W Zeng F Shi J Li J. HMGB1 promotes the release of sonic hedgehog from astrocytes. Front Immunol. (2021) 12:584097. 10.3389/fimmu.2021.584097 33868221 PMC8047406

[130] Becker KA Halmer R Davies L Henry BD Ziobro-Henry R Decker Y. Blockade of experimental multiple sclerosis by inhibition of the acid sphingomyelinase/ceramide system. Neurosignals. (2017) 25:88–97. 10.1159/000484621 29131010

[131] Chu Y Jing Y Zhao X Wang M Zhang M Ma R. Modulation of the HMGB1/TLR4/NF-κB signaling pathway in the CNS by matrine in experimental autoimmune encephalomyelitis. J Neuroimmunol. (2021) 352:577480. 10.1016/j.jneuroim.2021.577480 33493985

[132] Paudel YN Angelopoulou E Semple B Piperi C Othman I Shaikh MF. Potential neuroprotective effect of the HMGB1 inhibitor glycyrrhizin in neurological disorders. ACS Chem Neurosci. (2020) 11:485–500. 10.1021/acschemneuro.9b00640 31972087

[133] Grad LI Rouleau GA Ravits J Cashman NR. Clinical spectrum of amyotrophic lateral sclerosis (ALS). Cold Spring Harb Perspect Med. (2017) 7:a024117. 10.1101/cshperspect.a024117 28003278 PMC5538408

[134] Lee JD Liu N Levin SC Ottosson L Andersson U Harris HE. Therapeutic blockade of HMGB1 reduces early motor deficits, but not survival in the SOD1G93A mouse model of amyotrophic lateral sclerosis. J Neuroinflammation. (2019) 16:45. 10.1186/s12974-019-1435-2 30782181 PMC6380064

[135] Paudel YN Angelopoulou E Piperi C Othman I Shaikh MF. Implication of HMGB1 signaling pathways in amyotrophic lateral sclerosis (ALS): from molecular mechanisms to pre-clinical results. Pharmacol Res. (2020) 156:104792. 10.1016/j.phrs.2020.104792 32278047

[136] Lo Coco D Veglianese P Allievi E Bendotti C. Distribution and cellular localization of high mobility group box protein 1 (HMGB1) in the spinal cord of a transgenic mouse model of ALS. Neurosci Lett. (2007) 412:73–7. 10.1016/j.neulet.2006.10.063 17196331

[137] Lee JD McDonald TS Fung J Woodruff TM. Absence of receptor for advanced glycation end product (RAGE) reduces inflammation and extends survival in the hSOD1G93A mouse model of amyotrophic lateral sclerosis. Mol Neurobiol. (2020) 57:4143–55. 10.1007/s12035-020-02019-9 32676989

[138] Lee JY Lee JD Phipps S Noakes PG Woodruff TM. Absence of toll-like receptor 4 (TLR4) extends survival in the hSOD1 G93A mouse model of amyotrophic lateral sclerosis. J Neuroinflammation. (2015) 12:90. 10.1186/s12974-015-0310-z 25962427 PMC4431460

[139] Brambilla L Martorana F Guidotti G Rossi D. Dysregulation of astrocytic HMGB1 signaling in amyotrophic lateral sclerosis. Front Neurosci. (2018) 12:622. 10.3389/fnins.2018.00622 30210286 PMC6123379

